# Comparative Triceps Surae Morphology in Primates: A Review

**DOI:** 10.1155/2011/191509

**Published:** 2011-07-28

**Authors:** Jandy B. Hanna, Daniel Schmitt

**Affiliations:** ^1^Department of Biomedical Sciences, West Virginia School of Osteopathic Medicine, Lewisburg, WV 24901, USA; ^2^Department of Evolutionary Anthropology, Duke University, Durham, NC 27708, USA

## Abstract

Primate locomotor evolution, particularly the evolution of bipedalism, is often examined through morphological studies. Many of these studies have examined the uniqueness of the primate forelimb, and others have examined the primate hip and thigh. Few data exist, however, regarding the myology and function of the leg muscles, even though the ankle plantar flexors are highly important during human bipedalism. In this paper, we draw together data on the fiber type and muscle mass variation in the ankle plantar flexors of primates and make comparisons to other mammals. The data suggest that great apes, atelines, and lorisines exhibit similarity in the mass distribution of the triceps surae. We conclude that variation in triceps surae may be related to the shared locomotor mode exhibited by these groups and that triceps surae morphology, which approaches that of humans, may be related to frequent use of semiplantigrade locomotion and vertical climbing.

## 1. Introduction

From Aristotle's thoughts in *De Motu Animalium *[1], to Borelli's [2] comprehensive review of biomechanics in the 1600s, to Muybridge's [3] original documentation of gaits in horses around the turn of the last century, animal movement has been a vibrant and productive area of research providing insights into critical aspects of form-function relationships and selection pressures on limb and body design in many vertebrates, including primates. In addition to capturing photographic plates of the different gaits of the horse, Muybridge also took stop motion images of other mammals, including a nonhuman primate. His famous collection of plates and prose, published in 1887, was the first available to researchers interested in animal locomotion [3] and spawned a new generation of scientists interested in locomotion. Following Muybridge's observation that the baboon “disregards the law governing the walk” (3 : 30), multiple researchers created hypotheses concerning primate locomotor evolution, which were based on observed differences between primate and nonprimate locomotion (e.g., [4, 5]). For example, building on Muybridge's observations on footfall, in which he argued for a differential functional role of the forelimb and hindlimb in primates, forty years later, De la Croix [6] commented on the unique aspects of what he called “the pithecoid gait” (6 : 53 and Figure 3 therein), which he argued was “the gait used by the early ancestors of man.” (6 : 53, referring to [7]).

These early scientists laid the foundations for research in primate locomotor evolution. Since then researchers have been compiling a long list of features that distinguish the walking gaits of most primates from those of most other mammals. These features include the use of a diagonal sequence footfall pattern (e.g., [8–16]), the lack of a running trot with the use of an amble instead (e.g., [17–22]), relatively high hindlimb peak vertical forces (e.g., [16, 23–26]), highly protracted arms at touchdown (e.g., [27, 28]), and a deeply yielding elbow [29, 30]. These locomotor characteristics are hypothesized to have been important for the evolution of a diverse array of locomotor modes (e.g., [5, 25, 31–34]), but most of these locomotor modes appear to have been facilitated by a basal differentiation of the functional role of the forelimb and hindlimb.

It was first argued by Jones [5] that adaptations to moving and foraging on arboreal supports required the “emancipation of the forelimb,” leaving to the hindlimb the “servile” function of weight support. Jones [5] thought, as later experimental data would support, that primates were unusual among mammals in the different functional roles of the forelimb and hindlimb. Cartmill's work [35, 36] suggests that the earliest ancestors of primates were adapted to move and forage in a fine-branch arboreal milieu and that mechanical changes in forelimb use would have provided advantages in such an environment. This argument was supported later by studies of primates and arboreal opossums [12, 13, 25, 37, 38]. The evolution of later locomotor specializations including suspensory locomotion, vertical clinging and leaping, and bipedalism may have been facilitated by the reduced role in compressive weight support for the forelimb [5, 29, 31, 39, 40]. Thus, from this perspective it seems reasonable to argue that primate locomotor evolution is characterized by dramatic changes in the functional role of the forelimbs. Rather than have a near-equal division of labor between forelimbs and hindlimbs as in almost all other legged vertebrates, primates have exhibited a change such that we might describe them as hindlimb dominated relying heavily on the hindlimbs to power locomotion [23]. The forelimbs of primates, in contrast, may be described as “free” to provide stability and guidance (“steering”) as well as grasping and manipulation ([23]; see also [24, 26] for a more nuanced consideration). This changed functional relationship between the forelimb and hindlimb is highlighted in the many ways in which the walking gaits of primates differ from those of other mammals. This pattern appears to relate to the biomechanical challenges of arboreal locomotion and reflects a forelimb used less in compressive weight support and more in complex movement of guidance and manipulation. A number of specific analyses support this argument.

Vertical peak force (Vpk) data during quadrupedal locomotion support the claim that primate fore- and hindlimbs are functionally differentiated (e.g., [23–26, 29, 32, 33, 41, 42]). Additional work indicates that fore- and hindlimb differentiation is present in some primates during locomotor modes other than quadrupedalism (e.g., [43–46]). For reasons related to the evolution of primate locomotion and especially the evolution of bipedalism, much of the work done to date concentrates on the forelimb and to some extent the hip and thigh. The lower limb, and especially the leg, has received less attention. It is the goal of this chapter to better understand the functional anatomy of the leg in primates. In this light, in order to better understand this functional differentiation and how it is reflected in anatomical features of the lower limb, this paper reviews the functional morphology of the locomotor apparatus of primates, with a special emphasis on the leg, and places those data in a functional and evolutionary context.

The functional morphology of the primate forelimb has been well documented. In primates, the forelimbs appear to be relatively more mobile than the hindlimbs, particularly at the shoulder [33], the forelimbs have a different distribution of bone relative to the hindlimb and nonprimate fore- and hindlimbs [47], and hand morphology is very specialized (e.g., [35, 36, 48–53]). 

Additionally, the primate hindlimb presents morphological differences (compared to nonprimates) related to the differentiation of the fore- and hindlimb. When the hindlimb is considered it is often discussed in its relationship to leaping (e.g., [54–59]) and bipedalism (e.g., [60–62]). Therefore, rather than focusing on locomotion and manual manipulations as researchers have when discussing the forelimb, research on the functional morphology of the hindlimb focuses on the changes the hindlimb underwent to become the main organ of support and propulsion during locomotion. Various investigations of the pelvis, hip, and thigh document the morphological variation and functional morphology of the musculature of primates (e.g., [55, 63–67]). However, relatively few studies have been conducted on the leg (e.g., [63, 67–71]). This dearth of research on the evolution of the leg in primates is surprising because during human locomotion, the plantar flexors are important in providing propulsion and stability [72, 73]. Specifically, the triceps surae muscles are a large source of power for forward propulsion during human bipedal locomotion (e.g., [74–76]) and important during quadrupedal progression (although less than during bipedalism) (e.g., [77–79]). Although much has been described concerning the comparative anatomy of the primate hip and thigh muscles with reference to bipedalism, the comparative anatomy of triceps surae has been understudied. 

Numerous qualitative descriptions of the triceps surae for nonhuman primates (and other mammals) indicate notable departures from the morphology of these muscles in humans (e.g., [71, 80–82]). These morphological differences may be related to differences in locomotor mode. Understanding the nature of the triceps surae across taxa and across locomotor modes may provide a better insight into the diversification of primate locomotion and help illuminate the functional and adaptive patterns that lead to variation in leg muscles. 

Thus, the goal of this paper is to understand to what extent there is variation in triceps surae across species. We also ask to what extent that variation, when it exists, occurs in a pattern that allows for functional interpretation such as increased force production, excursion, or velocity (function). Finally, we ask if functional interpretations, when possible, are reflective of an important role in the animal's ecology (biological role). The next section begins by defining and summarizing the qualitative literature on the triceps surae in mammals. 

## 2. Methods

This paper is a literature review. Data on the functional morphology of the triceps surae (TS) were compiled from the literature and compared across a variety of primates and a few, nonprimate species. Much of the literature concerning TS is qualitative; thus, the section depicting the initial results presents those descriptions. Subsequent to that, quantitative data on fiber type distribution and relative mass of the muscles composing TS are presented. Finally, possible patterns of TS morphology are discussed with reference to literature regarding the biomechanics of movement. The literature compiled and presented includes data from both wild and captive animals, dry and wet weights of the muscles, and animals of known and unknown ages. To attempt to account for some of the differences likely caused by the lack of controls, the fiber type and mass data are presented as percentages, relative to the total percent of the individual muscle (in the case of fiber type distribution) or the total mass of TS (in the case of percent mass). Despite these attempts for better control, data should be interpreted with caution. 

### 2.1. Definition of Triceps Surae

Generally, comparative anatomists have defined triceps surae as the two heads of gastrocnemius and soleus. This definition is based on the human condition of triceps surae because, as Frey [70] points out, triceps surae is not always composed of three muscle bellies in mammals. Additionally, plantaris is almost always discussed in concert with gastrocnemius and soleus, although it is not technically considered part of triceps surae in standard, human anatomical descriptions [83]. Therefore, this paper will discuss both heads of gastrocnemius, soleus, and plantaris, and include all four of these muscle bellies under the general term triceps surae. 

Gray describes gastrocnemius, soleus, and plantaris in humans as follows [83]. Medial and lateral gastrocnemius are the most superficial muscles of the calf, taking origin from the condyles of the femur and inserting into the calcaneus. Plantaris is defined as the next most superficial muscle (lying between gastrocnemius and soleus), with an origin on the lateral side of the femur along the supracondylar line; plantaris is absent in approximately 5–10% of the population [83, 84]. Plantaris' insertion is normally into the calcaneal tendon, although occasionally variations occur. Finally, soleus is the deepest muscle of TS (e.g., the most anterior), taking its origin from the proximal fibula and middle third of the proximal tibia, along the soleal line; soleus inserts into the calcaneus via the calcaneal tendon. 

## 3. Results

### 3.1. Qualitative Descriptions of Triceps Surae in Mammals

Most other mammals' arrangements of TS are in contrast to the human condition. Lewis [85–87] provides a comprehensive review of the evolution of the cruropedal flexor musculature of the foot. In his reviews, he points out that marsupials—possessing the primitive condition for mammals—exhibit a plantaris, two heads of gastrocnemius, and no soleus. In marsupials, the lateral head of gastrocnemius arises from the lateral femoral condyle and is usually associated with a large fabella (a sesamoid bone posterior to the femoral condyles, within the gastrocnemius tendons) from which plantaris arises; the medial head of gastrocnemius originates similarly on the medial side of the femur [68–70, 85–88]. Both heads of gastrocnemius end in intertwined tendons and insert onto the calcaneus, while plantaris terminates in a tendon that generally expands into the plantar aponeurosis. Argot [89] suggests that gastrocnemius and plantaris are the largest muscles of the leg in highly terrestrial marsupials (8% of total hindlimb muscle mass) compared to highly arboreal species (4% of total hindlimb muscle mass). Frey [70] also indicates that soleus arises from the lateral head of gastrocnemius and is not well developed and even absent, in many marsupials. These data are contra Glaesmer [68], who suggested that soleus arises from the medial head of gastrocnemius. Later anatomists support Frey's description of a lateral derivation of soleus, when present in marsupials (e.g., [71, 87]). An analog to soleus in marsupials, however, is the flexor digitorum fibularis, which in highly arboreal marsupials is robust (9% total hindlimb muscle mass) [89]. 

The origin and insertion patterns of TS are slightly different in monotremes compared to marsupials. In these former species, soleus is a completely separate muscle (although still closely allied with gastrocnemius), taking its origin from the head of the fibula [70, 85]. The lateral fabella that gastrocnemius originates from is typically diminished and occasionally fuses with the fibula [70, 85]. 

In true placental mammals (Eutheria), plantaris and both heads of gastrocnemius originate from the femur, although fabella may still be present in many taxa [68–70]. In some primates, plantaris inserts into the heel instead of the plantar aponeurosis [88], and in some groups of mammals, plantaris is referred to as flexor digitorum superficialis [90, 91]. Soleus is variably present in other orders of mammals (e.g., [70, 71, 85–87, 90–92]). When it is present, its origin is typically limited to the fibula.

**Figure 1:**
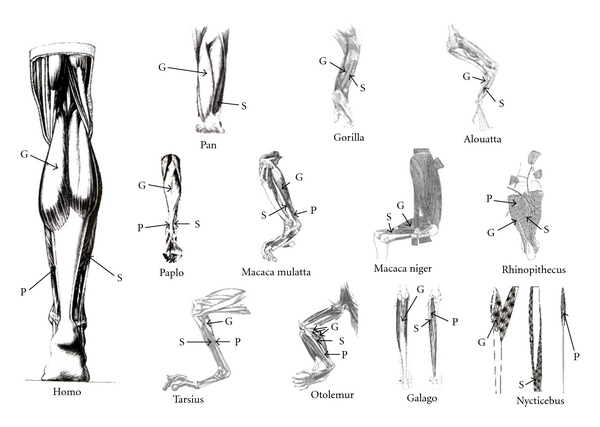
Illustrations of the superficial leg musculature of various primate species. Note that plantaris is larger than in the typical human condition in most images (e.g., *Galago, Otolemur, Tarsius, Macaca*, and *Rhinopithecus*). Species in which plantaris is similar in size to the human condition are *Gorilla*, *Pan*, and *Alouatta*. Image adapted from: (1) 95-*Otolemur* (published as *Galago crassicaudatus*), (2) 94-*Tarsius*, (3) 96-*Macaca niger* (published as *Cynopithecus niger*) and *Rhinopithecus*, (4) 80-*Macaca mulatta*, (5) 81-*Alouatta*, (6) 98-*Galago*, (7) 175-*Gorilla*, (8) 99-*Nycticebus* and (9) 97-*Homo*, *Pan*, and *Papio*. “G” indicates gastrocnemius, “S” indicates soleus, and “P” indicates plantaris.

It appears, therefore, that the broad mammalian pattern of TS muscle anatomy is to have a strongly developed lateral gastrocnemius, a somewhat less strongly to equally developed medial gastrocnemius, a substantial plantaris, and a weakly developed soleus (if present) [87]. However, according to various atlases, primates exhibit some variation in the degree of development of TS muscles. For example, Frey [70] notes ape morphology includes a much reduced plantaris and a more strongly developed soleus. Additionally in apes, plantaris inserts into the calcaneus instead of the plantar aponeurosis and medial gastrocnemius is more substantially developed than lateral gastrocnemius [82, 93]. Hartman and Straus [80] report the opposite morphology for the macaque, that is, a weak soleus and strong plantaris, and Woollard's [94] description of a tarsier suggests a pattern similar to monkeys and lemurs, as well. Descriptions of other species of primates illustrate the variation in TS morphology, as well (e.g., [52, 71, 81, 95–97]) (Figure 1). These illustrations suggest that chimpanzees have shorter tendons than humans, baboons, [97], macaques [80], and galagos [98, 99] (Figure 1). Additionally, dissection pictures suggest that lorisines also have a relatively short tendon [99]. 

### 3.2. Quantitative Measures of Triceps Surae Morphology

The previous section outlined qualitative descriptions of muscle variation. However, one of the problems with these descriptions is that the language used to describe the muscles is variable. For example, Hartman and Straus [80] describe the macaque plantaris as “strong and fleshy” (80: 159), while Woollard [94] describes the plantaris of the tarsier as “quite large” (94: 1175). In order to determine whether there are reliable differences in TS morphology, quantifiable traits are necessary. Ideally, such traits will have functional significance as well. Two commonly reported muscle traits are fiber type and muscle mass, although there are additional aspects of muscle structure, which may be informative about muscle function (e.g., fiber length, pennation angle, etc.). 

Muscle fiber type is related to the contractile properties of the muscle (the speed of contraction and the muscle's fatigability) and fiber type may be indicative of whether a muscle's main function is related to posture, as compared to movement. Muscle mass, specifically as a surrogate for cross-sectional area and fiber length, is representative of its ability to produce force, achieve work, and generate power (e.g., [100–103]). The following sections report muscle fiber type and mass for TS in primates, with some nonprimate mammalian species for comparison, as these variables are often reported as quantitative values in the literature. These following sections are not meant to be exhaustive of the known literature, and apologies are given to researchers whose data have not been included. 

### 3.3. Fiber Type Differences

For this paper, the simple “three-fiber” classification is used (slow-twitch oxidative (SO), fast-twitch oxidative glycolytic (FOG), and fast-twitch glycolytic (FG)). We recognize that this classification of fibers into three distinct groups is an oversimplification [104], but this schema delineates fiber type by relatively major differences in oxidative capacity, and most of the literature available is able to be grouped into this schema. For literature presenting additional fiber types on the basis of oxidative capacity, an arbitrary cut-off was made to classify type IIA and IIB fibers as FG and FOG, respectively, while type IIC were classified as FG. Including IIB fibers as FG instead of FOG does not affect the distribution of percentages to a large degree. This method increases the comparative sample and is valid because oxidative capacity is plastic [105]. Fiber type data are presented in the “three-group” classification (Table 1). 

Comparisons of muscle fiber types among species do not show large-scale differences. As expected, differences are generally found in the oxidative capacity among fast fibers. Much of the variation in fiber type may also be due to the sampling method, as it is impossible to know if each sample was taken from the same area and depth of the muscles. Various researchers have found that individual muscles tend to have fiber types organized stratigraphically. Specifically, oxidative capacity of fibers in muscle tends to increase in deeper layers (e.g., [111, 115–118]). This fact tends to confound comparisons based on the literature if the depth at which the fiber sample was taken is unknown. However, the lack of large interspecific differences in fiber type suggests that functional differences in animals (e.g., activity pattern or force production) are not related to fiber type. 

For all species, the large number of SO fibers in soleus (Table 1) suggests that this muscle is equipped for force production over long periods. Fatigue-resistant fibers would allow for prolonged activity (relative to glycolytic fibers), as is seen in electromyography (EMG) of the soleus during normal standing in humans [119]. In contrast to soleus, both heads of gastrocnemius have a majority of fast fibers, although the oxidative capacity of these fibers varies among species (Table 1). However, the lack of major differences between twitch fiber types suggests that any activity pattern or force production differences are more functionally related rather than fiber type related. Finally, similar to gastrocnemius, plantaris has a majority of fast fibers, although the oxidative capacity of these fibers varies (Table 1). The tendency is for plantaris to have a majority of fast glycolytic fibers in all species, but the difference in oxidative capacity is less acute in primate species. Additionally, while still possessing a majority of fast fibers, the primate plantaris tends to have a higher percentage of slow fibers than other mammalian species. Admittedly, data for only three nonprimate species are presented, two of which are rodents. Therefore, interpretations of these data should be cautious.

In summary, drawing strong conclusions from the fiber type data are not warranted. The strongest conclusion is that few acute differences exist in fiber types, particularly twitch-types, of specific muscles among species. Thus, activity pattern and force distribution differences are probably related to other contractile properties of the muscles, such as mass or volume. 

### 3.4. Muscle Mass Differences

Body mass has an important influence on the size of muscles. If a muscle is required to move a given load a set distance, it will be required to produce more force to move a heavier load a comparable distance (assuming moment arms are not changed). In order to accomplish this, a muscle's cross-sectional area must be increased. All other things being equal (e.g., muscle fiber length stays constant), an increase in cross-sectional area will be reflected as an increase in muscle mass. However, the scaling relationship of TS muscles (relative to body mass) in primates is unclear. For example, Alexander and colleagues [120] found that TS muscle mass scales at 1.27 relative to body mass (positively allometric) in a sample of primates, but Pollock and Shadwick [121] reported that muscle mass of plantaris and gastrocnemius in various mammals scales nearly isometrically (~0.90). Because of the unclear allometric relationship TS muscle mass has with body mass (or phylogeny), data are presented as percent contribution to total TS mass rather than relative to body mass (Table 2). This method of data presentation decreases the likelihood of interspecific differences being size dependent. 

Compared to fiber-type data, differences in muscle mass present clear patterns of variation (Table 3). In nonprimates, gastrocnemius mass comprises 65–80 percent of triceps surae. Plantaris follows next in mass, contributing 12–25 percent to TS mass. Finally, soleus is the smallest muscle of TS, generally contributing less than 10 percent to total TS mass. Bears and elephants, however, do not follow this pattern (Table 2). Strepsirrhines have a distribution of mass within triceps surae similar to nonprimates because plantaris is still relatively larger (10–20 percent of TS mass) than soleus. On the other hand, the strepsirrhine soleus contributes more mass to TS than in nonprimate mammals (10–20 percent). Gastrocnemius generally contributes between 65 and 75 percent to TS. There are two exceptions to the general strepsirrhine pattern of a relatively large plantaris compared to soleus. *Varecia* plantaris and soleus contribute equally to TS mass, and in lorisines, soleus contributes a much larger percent to TS mass than plantaris. The lorisine distribution of TS mass is similar to the human condition in having a relatively large soleus and relatively small plantaris.

In anthropoids, soleus contributes more mass to TS than plantaris. In cercopithecoids, plantaris constitutes about 10 percent of TS, similar to nonprimates, whereas soleus constitutes 24–30 percent of TS. Gastrocnemius correspondingly contributes slightly less to TS mass, averaging about 60–65 percent. In the platyrrhine species available (the howler monkeys), plantaris is not present and soleus and gastrocnemius contribute 45–55 percent, respectively, to TS. Nonhuman great apes have a similar pattern of muscle mass distribution to the howlers (and humans), although plantaris is variably present. When plantaris is present, its mass has not been reported consistently, suggesting a minimal contribution to the mass of TS. Finally, in humans, soleus is the largest muscle in TS, contributing over 60 percent of muscle mass to TS. Plantaris, as in nonhuman apes, lorisines, and atelines, appears to contribute little to TS mass.

Additionally, humans and gorillas are reported to have a larger medial head of gastrocnemius relative to the lateral head, while chimpanzees are reported to have symmetric heads of gastrocnemius [82, 93]. However, Table 2 shows that the chimpanzee actually exhibits asymmetry between the medial and lateral heads of gastrocnemius. Additionally, dissection illustrations and photographs support this asymmetry in chimpanzees [97] and show that lorisines also exhibit asymmetry in the heads of gastrocnemius [99] (Figure 1). Sonntag [135], although he presents no muscle mass data, reports equal-size heads of gastrocnemius in the orangutan but also states that the chimpanzee and gorilla exhibit asymmetry.

These data suggest that gastrocnemius produces the greatest force in TS in all primate species except humans. Medial gastrocnemius may be more important to force production than lateral gastrocnemius in some primates. Soleus becomes increasingly important as a force producer in anthropoids (compared to other mammals) and eventually takes over the role of primary force producer in humans. Soleus appears to contribute more to force production in great apes, atelines, and lorisines than in other nonhuman primates. Plantaris is not an important force producer in humans, other apes, atelines, and lorisines, although it remains important for locomotion in Old World monkeys. 

## 4. Discussion

This sample is, at best, limited and there are some questions that remain unanswered. First is how does phylogeny affect TS variation? Although Langdon [71] suggested that there is a “typical TS morphology” within primate phylogenetic groups, the sample does not clearly illustrate this suggestion. Additionally, in other mammalian groups, similar variation within phylogenetic groups is present. For example, within Carnivora, the black bear is described as having a well-developed soleus and *three* heads of gastrocnemius, but no plantaris [136], while the polar bear is reported to have only a plantaris and two heads of gastrocnemius [137]. Finally, Ray [138] reports the presence of all four muscles in the Malay bear, with plantaris being approximately the same size as the lateral gastrocnemius. In another group of carnivores, the cat has a large plantaris and a small soleus, while canids have no soleus at all [85, 90]. Similar diversity in TS morphology is exhibited in other species, such that African elephants exhibit a robust soleus and limited plantaris [139], while hippopotami lack a soleus [91, 140], but exhibit a “fleshy” plantaris [91]. 

Second, how does the age of the individuals impact these data and what were the ages of the individuals studied? Unfortunately, all the mass data should be interpreted with caution because the age of many of the subjects is unknown. It may be that many of the subjects were not fully adult or did not exhibit locomotor mode usage similar to their wild counterparts. Both muscle mass and fiber type may not accurately represent the adult morphology because young primates do not always exhibit the same distribution of locomotor mode usage as adults (e.g., [141–144]). In particular, hindlimb mass characteristics change more dramatically during ontogeny [142]. 

Third, do atelines differ in their specific muscle mass contribution to TS compared to other New World monkeys, as suggested by the qualitative literature [145]? The current sample, unfortunately, consists of only one genus of New World monkey, the howler monkeys, and its TS mass data include plantaris with gastrocnemius [63, 128]. It is assumed that this is because plantaris is not completely distinct from gastrocnemius, as several researchers have previously observed (e.g., [81, 145]). Therefore, the data presented here cannot confirm or reject the hypothesis that atelines differ in their TS morphology compared to other New World primates. Additionally, dissections of *Pithecia, Aotus, *and *Saimiri *suggest that plantaris is very small in these species as well (pers. obs.). *Saguinus rosalia* is reported to have missing plantaris by Windle [146], whereas others have reported a distinct plantaris in callitrichids [145]. 

These conflicting data on the condition of TS in platyrrhines present a problem. The current data suggest that both hominoids and at least some platyrrhines share a similar TS morphology, possibly indicating this morphology is phylogenetically based, that is, basal to anthropoids. It is possible that the cercopithecoid condition is a derived feature relative to the anthropoid state. However, the fact that lorisines also share the hominoid and platyrrhine (or ateline) TS condition suggests that TS morphology has evolved independently in these three groups, indicating some type of functional convergence.

In summary, muscle fiber type data appear to be similar across species, while muscle mass data suggest three separate patterns (Table 3). The general mammalian pattern of mass distribution in TS in which the order from largest to smallest (and therefore, in order of importance to force production) is gastrocnemius, plantaris, and soleus (when present). Prosimians, exclusive of lorisines, share this pattern of mass distribution in the TS. However, anthropoids exhibit a different pattern of mass distribution, with gastrocnemius still the largest contributor to TS mass, soleus the second largest contributor, and plantaris contributing the least mass to TS. Additionally, in anthropoids, the relative mass contribution of plantaris is not reduced the relative mass of gastrocnemius, however, is reduced. Nonhuman great apes, atelines, and lorisines all exhibit the third pattern of muscle mass distribution in TS in which the gastrocnemius is the largest contributor to total TS mass, followed closely by soleus, and finally with plantaris (when present) contributing only 6 percent or less to the mass of TS. Additionally, limited data suggest asymmetries in muscle mass between medial and lateral gastrocnemius in primates, with the typical pattern being a larger lateral head, although apes and lorisines appear to possess a larger medial head of gastrocnemius. Finally, the muscle-tendon ratio of TS appears to be different in great apes and lorisines compared to other mammals. It is possible that the shared morphology of non-human great apes, atelines, and lorisines is a functional convergence. The remainder of the discussion explores that possibility by examining various characteristics of locomotor mode, such as muscle activity pattern, pressure, and force data, and joint kinematics. 

### 4.1. EMG Pattern and Kinematics during Locomotion

The EMG pattern of muscles during locomotion is useful for determining the duration of force production. These data, in combination with kinematic data, are important for understanding the role a muscle has during a specific movement. In this next section, we discuss experimental data on activity pattern and kinematics related to triceps surae in nonprimate mammals and primates. 

### 4.2. EMG: Nonprimate Quadrupedalism

Most EMG work done on leg muscles during quadrupedal locomotion has examined the cat and the dog (e.g., [147–151]). The activity of triceps surae during quadrupedal locomotion in these species is similar, although limited data on all TS muscles exist. In the dog, gastrocnemius is active right before touchdown and extends through touchdown, eventually tapering off about half way through stance phase; there is limited activity of gastrocnemius during toe-off [148]. Tokuriki [148] interprets this pattern as stabilizing the ankle (and knee) against the substrate reaction force. Similarly, Engberg and Lundgren [147] found that the gastrocnemius in the cat is active right before touchdown and activity continues slightly past midstance phase. They interpret this activity as lowering the foot to place the pads on the ground (because the cat is digitigrade) and then resisting passive dorsiflexion. As in the dog, there is limited activity during toe-off. The cat soleus and plantaris have activation patterns similar to those recorded for gastrocnemius, although soleus tends to become active before gastrocnemius and is relatively more active throughout stance phase [149, 151]. EMG data are available for other species of nonprimates, such as the guinea pig and rat, and the activity pattern of TS muscles is generally similar to the cat and the dog (e.g., [152, 153]). 

Summarizing the nonprimate data on activity pattern during quadrupedal locomotion suggests that both gastrocnemius and soleus are important in producing force during stance phase, although force production in gastrocnemius may stop midway through stance phase. This force production is important for stabilizing the ankle and producing propulsive thrust at toe-off. Nonprimate mammals appear to use TS to lower the foot to the ground at touchdown, as well. Admittedly, few species are available for a robust comparison. 

### 4.3. EMG: Primate Quadrupedalism

For quadrupedal walking in primates, few EMG data on the leg muscles have been collected. Kimura et al. [23] presented EMG data for gastrocnemius in six species of nonhuman primates during quadrupedal walking. Japanese macaque EMG was similar to cats and dogs, while the spider monkey EMG differed slightly. Hodgson and colleagues [154] corroborate Kimura and colleagues' [23] data by finding that EMG data on medial gastrocnemius of the macaque is similar to cats and dogs. Like nonprimate mammals and most primates, the spider monkey gastrocnemius becomes active at the beginning of stance phase; however, in this species this activity is weak. Unlike dogs, cats, macaques, and other primates, strong activity is found late in stance phase. Kimura et al. [23] attribute this activity to the greater weight borne on the hindlimbs by the spider monkey. Additionally, EMG data collected by Kimura et al. [23] show gastrocnemius activity in the chimpanzee is similar to the spider monkey, while baboon gastrocnemius EMG is more similar to the Japanese macaque. EMG data on soleus in the rhesus macaque show activity throughout stance phase, as well [107]. 

### 4.4. EMG: Bipedalism

Most muscle activity pattern during locomotion has been collected on humans, but there are EMG data for nonhuman primates during bipedal locomotion (discussed below). This section presents TS activity pattern data during human bipedalism first and then goes on to discuss nonhuman primate EMG data during bipedalism. Because plantaris is so small (and sometimes absent) in humans, few EMG data are available for it. O'Connell [155] conducted experiments on triceps surae, including plantaris, but did not present any results for plantaris. Soleus and gastrocnemius, on the other hand, have been studied in more detail. Soleus becomes active just after touchdown and continues throughout stance phase; its activity stops before the hallux clears the ground [72, 156, 157]. This pattern has traditionally been interpreted as activity for support rather than propulsion (the idea being that the ankle plantar flexors must resist passive dorsiflexion during stance phase [158]). However, given the latency of force production relative to EMG recording [159, 160], it can be argued that soleus probably contributes to propulsive force production, specifically of the trunk, at toe-off, an idea supported by recent experimental work (e.g., [161, 162]). Gastrocnemius activity commences just before touchdown and stops until activity recommences just before toe-off [163]. Other studies (e.g., [164, 165]) have shown that activation of TS muscles depends on the speed of locomotion, with early activation of gastrocnemius occurring only at higher speeds. Nilsson et al. [165] did not find gastrocnemius active at touchdown during walking, but did find it active at touchdown during running. Mann and Hagey [164] found a similar discrepancy in gastrocnemius activation during walking and running. This early activation of gastrocnemius may be related to lowering the foot to the ground during running (similar to cat and dog kinematics), as some humans tend to plantarflex the foot during running, causing touchdown to occur at the forefoot (e.g., [166–169]).

Activity patterns of triceps surae in some nonhuman primates encouraged to walk bipedally sometimes differ from the patterns exhibited during quadrupedalism in the same species. In fact, it has been suggested that triceps surae are much more important for balance and propulsion during bipedal locomotion than during quadrupedalism in nonhuman primates [73]. Ishida et al. [170] present EMG data for the lateral head of gastrocnemius for 6 primate species, including humans. While the baboon, macaque, and chimpanzee exhibit muscle activity similar to EMG data collected during quadrupedalism in the same species, the spider monkey and gibbon exhibit a two-peak pattern of EMG activity [170]. The first peak occurs at touchdown, while the second occurs during the latter half of stance phase, similar to the single peak found in the spider monkey during quadrupedalism [23]. Ishida and colleagues' [171] additional EMG data for the gibbon gastrocnemius show the two-peak activity of gastrocnemius found in the earlier study. Although Ishida et al. [170] suggest that the spider monkey bipedal gait is similar to the chimpanzee, which exhibits heel strike, it is suggested that the spider monkey does not exhibit heel strike during bipedal locomotion. In fact, the spider monkey does not exhibit heel strike during quadrupedal locomotion [172], so it is probable that the two-peak EMG activity of the gastrocnemius of the spider monkey during bipedal locomotion is related to initial contact during stance phase being made by the phalanges and metatarsals, similar to the toeing down of the gibbon during bipedal locomotion [173]. Indeed, Okada [174] supports this conclusion. This action is similar to the toeing down of digitigrade species during quadrupedalism and therefore should exhibit a similar EMG pattern. The second peak of EMG activity of gastrocnemius for the gibbon and spider monkey is similar to quadrupedal EMG pattern in the spider monkey. This similarity may be maintained by similar kinematic patterns of the leg during both modes of locomotion. Additionally, Ishida et al. [171] collected data on the gibbon soleus during bipedalism. They show that soleus reaches peak activity during the latter half of stance phase, although it is active throughout stance. 

### 4.5. EMG: Vertical Climbing

EMG activity patterns of TS muscles during vertical climbing for the Japanese macaque are similar to those during quadrupedal and bipedal walking (most data are solely on gastrocnemius) [23, 170, 175]. Gastrocnemius peak activity is exhibited during the first half of stance phase, similar to the activity seen during quadrupedalism. The similarity in EMG activity pattern among locomotor modes suggests similarity in kinematic parameters as well. Indeed, the Japanese macaque tends to use flexed hip and knee postures during vertical climbing, and it uses minimal ankle excursion [44, 45, 175, 176]. These kinematic characteristics are similar to data during quadrupedal walking in the Japanese macaque [23, 172]. Additionally, kinematic data are available for the spider monkey during vertical climbing [44, 45, 175, 176]. In these studies, the authors found that the spider monkey tends to use more extended hindlimb postures at the hip and knee (compared to the Japanese macaque). Conversely, the spider monkey ankle goes through greater excursion during the limb contact phase and is generally more dorsiflexed throughout stance phase. These three joint angle characteristics are similar to those during human bipedalism (i.e., extended hip, extended knee, and large ankle excursion [170]). Hirasaki et al. [44] also found that the hindlimb contact time was absolutely and relatively (to the forelimb) longer in the spider monkey than in the Japanese macaque. This may be a consequence of the greater excursion of the limb [172]. 

### 4.6. Force Data and Kinematics

In addition to a review of available EMG data, force and pressure data are available which may correspond to the forces exerted by TS muscles. Force data are generally collected by having a subject move over a force plate instrumented with gauges that are sensitive enough to register the substrate strain caused by a body exerting force on the substrate. Force data are generally composed of three components, one vertical and two horizontal components: a braking-propulsive component and a mediolateral component. In addition to force data, pressure data can be collected in a similar manner using commercial devices [177]. Force and pressure data reflect to some degree the amount of force muscles must exert to produce movement. The following section reviews the available data on force and pressure recordings in primates during locomotion. 

#### 4.6.1. Force Data: Quadrupedalism

Although much of the literature on vertical peak force (Vpk) during primate quadrupedalism has suggested that most primates experience higher Vpk on the hindlimbs than on the forelimbs (e.g., [23–26]), the data show that only the chimpanzee, orangutan, and spider monkey experience a statistical difference in weight bearing between the fore- and hindlimbs [26]. Mediolateral force data collected from mammals during quadrupedal walking on the ground suggest that most mammals exert higher lateral than medial force by the hindlimbs [23, 24, 178, 179]. Several primates, however, exhibit higher medial forces on the hindlimbs, including the spider monkey [23] and chimpanzee [180]. Additionally, when primates move quadrupedally on an arboreal substrate, they exhibit greater medial forces [178, 179]. 

#### 4.6.2. Force Data: Bipedalism

Foot pressure data on chimpanzees during arboreal quadrupedalism and bipedalism suggest that greater pressure is borne medially (relative to laterally) [180]. Li et al. [181] found that during chimpanzee bipedalism, higher medial pressure is exhibited on the foot. Force data collected synchronously show higher forces medially, as well [181]. Data collected on humans walking with flexed hip and knee postures (Groucho walking) also show higher medial pressures and force similar in magnitude to that found during chimpanzee bipedalism [181]. Humans walking with extended postures exert even more pressure and force medially [181]. 

#### 4.6.3. Force Data: Vertical Climbing

**Figure 2:**
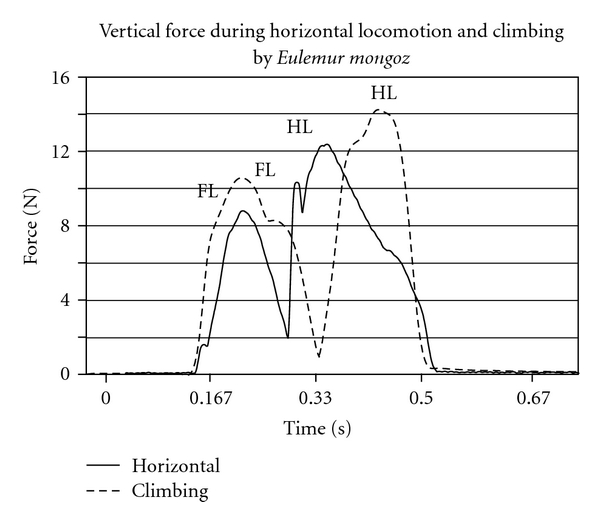
Representative vertical force traces during horizontal and vertical locomotion by the same individual of *Eulemur mongoz* (Hanna, unpub. data). The speeds are approximately similar in the two locomotor trials. Note that the forelimb peak forces during both horizontal locomotion and climbing are lower than the hindlimb peak forces.

Force data have been collected during vertical climbing in the Japanese macaque and the spider monkey [44, 45], for the long-tailed macaque [46] and for several lemurs and lorises [182]. These data show that the spider monkey experiences proportionally greater weight bearing by the hindlimbs than by the forelimbs when compared to macaques and lemurs. Macaques and lemurs (Figure 2) experience greater weight bearing by the hindlimb, but the magnitude of weight is not as great as that borne by the spider monkey. A third pattern is exhibited by lorises in which lorises experience greater forelimb forces than hindlimb forces during climbing. The force distribution during vertical climbing by the spider monkey (compared to the macaque and lemurs) suggests a greater reliance on the hindlimb (and, therefore, on TS to generate force to move the animal upward) than in the macaque and lemur [44, 45, 175, 176]. Further study confirms this hypothesis by showing that in the spider monkey, the hindlimb muscles (including gastrocnemius and soleus) can exert larger forces than the forelimb muscles [176]. In particular, Hirasaki et al. [176] suggest that “the spider monkey type of climbing could develop the hindlimb extensor muscles” (page 455). No pressure data are available during vertical climbing for these species.

Similar to the studies by Hirasaki et al., [44, 45, 175, 176], Yamazaki and Ishida [183] collected kinematic data and calculated joint moments and muscular force on the gibbon during vertical climbing. They found that gibbon TS exerts large moments at the ankle, suggesting that TS should be large in the gibbon. 

### 4.7. Morphological Patterns

The data summarized above suggest that primates differ from nonprimate mammals in several key ways and that one group of nonhuman great apes, atelines, and lorisines exhibit a particularly unusual pattern of TS anatomy. First, these taxa have relatively large soleus muscles compared to other taxa, and the percent contribution to TS mass by soleus is similar in these groups. Second, the relative mass of gastrocnemius and plantaris is reversed compared to other taxa. Third, great apes and lorisines may have a short Achilles tendon compared to other species. Fourth, nonhuman apes, and possibly lorisines, have a more massive medial head of gastrocnemius compared to the lateral head. If this asymmetry in mass between the heads of gastrocnemius is functionally related to locomotor mode, then atelines may exhibit a similar morphology. Finally, apes also occasionally exhibit a medial expansion of soleus. In this paper we propose that all these features are functionally related to vertical climbing or plantigrady. Specializations for both types of locomotion may relate to the evolution of bipedalism. 

### 4.8. Locomotor Data

#### 4.8.1. Quadrupedalism

The EMG data present some interesting patterns that may correlate with the observed muscle mass pattern discussed above. First, the EMG data suggest differences in TS activity pattern that correspond to digitigrade species (e.g., macaque, baboon, cat, and dog) and semiplantigrade species (e.g., spider monkey and chimpanzee (see [172] for a review of kinematics of these species). Semiplantigrade (or plantigrade) locomotion is defined here as any time the heel contacts the ground during stance phase. The semiplantigrade species exhibit longer and generally greater magnitudes of muscle activity than the digitigrade species, suggesting potentially higher force production by TS throughout stance and at toe-off in semiplantigrade species compared to digitigrade species.

If the difference in EMG activity is related to foot contact kinematics, the implications are threefold. First, an increase in muscle activity pattern in semiplantigrade species, both in magnitude and duration, is supported by the observation of Schmitt and Larson [172] suggesting that the heel of *Ateles* (and great apes) contacts the ground during quadrupedalism, although after initial foot contact. Second, greater muscle activity during toe-off in the plantigrade species suggests greater reliance on TS for propulsive force production. Finally, both these facts suggest TS mass, particularly the relative contribution of soleus, should be greater in semiplantigrade species. In primates, this conclusion is supported by TS mass data on chimpanzees (TS as a percentage of body mass = 0.77 [124]) compared to macaques (TS as a percentage of body mass = 0.47 [111]) (Table 2). Recent myological description of African elephant legs supports this hypothesis, as well, in that soleus is relatively large [139]. Myological data for bears also support this hypothesis (Table 2). If EMG data on plantigrade/semiplantigrade non-primate species (e.g., bears and elephants) were available and indicated that soleus is more active during toe-off than during digitigrade species, then a functional link could be drawn between the architecture of TS, particularly soleus, and activity pattern.

Additionally, kinematic data on digitigrade primates (macaque and baboon) versus semiplantigrade species (*Ateles *and chimpanzee) show that the semiplantigrade primates have greater protraction of the hindlimb [28, 30, 32, 172]. In these cases, the center of mass appears to be placed behind the foot [172], potentially further from the center of joint rotation. If the lever arm is longer in the spider monkey compared to the macaque, then more force would need to be exerted by the spider monkey TS to move the center of gravity. Therefore, a larger TS mass might be expected in the semiplantigrade species for this reason, as well.

Finally, chimpanzee and spider monkey kinematic data show that these species have longer hindlimb contact time than the macaque or baboon [172]. In species with correspondingly long contact times, muscle activity should be relatively extended. In these cases, muscles with a high proportion of fatigue-resistant fibers (e.g., soleus) should be utilized and potentially be more massive than muscles with a higher proportion of glycolytic fibers (e.g., plantaris and gastrocnemius). The large soleus of the chimpanzee (and potentially the spider monkey) relative to its plantaris may be functionally correlated with the long hindlimb contact times during quadrupedal locomotion.

Therefore, for several reasons, semiplantigrady should be correlated with larger TS mass and potentially a relatively large soleus, to meet the needs of greater and more extended force production during quadrupedal locomotion. In particular, high hindlimb protraction should be correlated with relatively large TS, and long contact times should be correlated with a large soleus. Unfortunately, activity pattern data cannot contribute to an understanding of the relative proportions of the individual TS muscles because most EMG data for nonhuman primates have been collected for gastrocnemius only. Finally, lorisines, which share a similar TS morphology with great apes and atelines, exhibit large degrees of hindlimb protraction [184], although they are not semiplantigrade. Thus, semiplantigrady alone cannot explain the presence of a large soleus. Rather, the functional explanation of a large soleus may be the combination of large hindlimb protraction and long contact times. 

#### 4.8.2. Quadrupedalism Compared to Bipedalism

The function of the muscles of TS in quadrupeds and bipeds is to support the body, resist external forces, and generate force for propulsive thrust. However, in some species, TS activity pattern in bipeds versus quadrupeds sometimes differs. Specifically, semiplantigrade species (e.g., chimpanzee and spider monkey) may have TS EMG activity patterns similar to humans, while other primate and nonprimate species appear to exhibit a different pattern of muscle activity. These two distinct EMG activity patterns suggest functional differences in the role of the muscles in question. In humans, great apes, and atelines, TS may produce more force over a longer time when compared to cats, dogs, or macaques. TS may also need to produce greater force in humans, great apes, and atelines due to kinematic parameters (e.g., long contact time and high protraction). These functional differences may be correlated with differences in muscle morphology, as humans, great apes, and atelines share a similar pattern of relative TS mass distribution (large soleus, small plantaris). The question then becomes as follows: what are humans, apes, and atelines doing during quadrupedal and bipedal locomotion that causes similar activity pattern of TS muscles? It is probable that the kinematics discussed above (highly protracted hindlimbs, semiplantigrady, and long contact time) contribute to the similarity in EMG activity pattern between bipeds and quadrupeds. 

#### 4.8.3. Bipedalism

During bipedal walking, it is hypothesized that the gibbon and spider monkey exhibit greater hindlimb excursion than the baboon or macaque, so that at toe-off, the ankle joint center is further away from the center of mass. Such kinematics would require greater activity of TS (gastrocnemius and soleus, although few data are available for soleus) at toe-off in the spider monkey and gibbon compared to the baboon or macaque and would therefore explain the similarity between human gastrocnemius EMG and spider monkey and gibbon gastrocnemius EMG (i.e., peak activity during the latter half of stance phase). Additionally, data on gibbon bipedalism suggest that the gibbon and human ankle excursion patterns are very similar [185]. These ankle excursion data suggest similar relative force production throughout stance in the gibbon and human by the TS muscles. 

#### 4.8.4. Vertical Climbing

**Figure 3:**
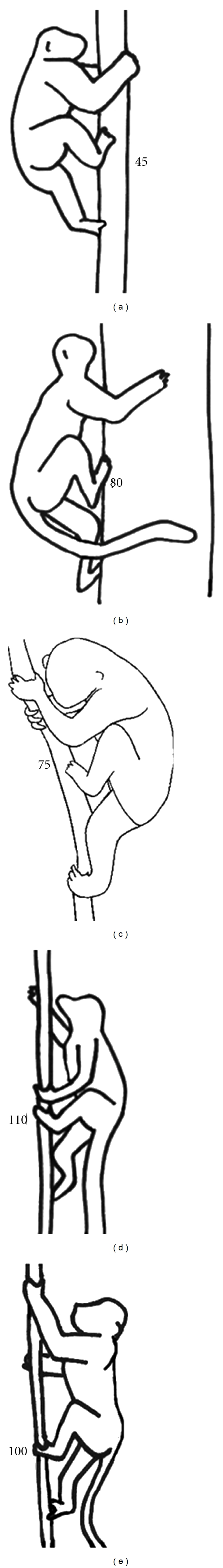
Representations of ankle joint during climbing by different primate species. The hindlimb foot most cranial has just touched down in the gait cycle. Dorsiflexion values at touchdown are reported next to the touchdown foot:** (**a) chimpanzee (image and dorsiflexion value (maximum) adapted from [189, 190]), (b) spider monkey (adapted from 168, dorsiflexion value from 45), and (c) slow loris (image adapted from 198, dorsiflexion value from Hanna, unpub. data). Note the highly dorsiflexed ankle position in these species (ankle angle < 90 degrees), (d) mongoose lemur (image and dorsiflexion value from Hanna, unpub. data), and (e) long-tailed macaque (image from Hanna, unpub. data, dorsiflexion value from 46). Note the less dorsiflexed position of the ankle in these species (ankle angle ≥ 90 degrees).

A variety of studies on vertical climbing in primates have been conducted, which suggest that differences in primate crural myology may be related to vertical climbing. For example, vertical climbing EMG on gastrocnemius in the spider monkey and gibbon is more similar to EMG of the same muscle during human bipedalism than during climbing by the Japanese macaque [44, 45, 175, 176, 183]. Additionally, force data during vertical climbing support a greater fore- and hindlimb differentiation and greater hindlimb joint excursions in the spider monkey and gibbon than in the Japanese macaque. Finally, kinematic data during vertical climbing are published for the spider monkeys [44, 45, 186], macaques [44–46, 175, 176], gibbons [183, 187, 188], great apes [188–190], *Rhinopithecus* [186], and lemurs and lorises [191]. These kinematic data suggest that great apes, atelines, and lorisines exhibit similar ranges of dorsiflexion during vertical climbing and are greater than those of lemurs and macaques (e.g., [45, 46, 124, 186, 187, 191]) (Figure 3). Data on dorsiflexion in *Rhinopithecus* are not reported [186]. Osteological measurements of the talocrural joint support the kinematic data in that great apes and spider monkeys (no data are available for lorisines) exhibit similar distal tibia and talocrural morphology related to the large degree of dorsiflexion exhibited during vertical climbing in these species [189, 190]. These data suggest that the convergent mass distributions of the TS muscles may be related to the range of dorsiflexion exhibited during vertical climbing in great apes, atelines, and lorisines. 

So far, great ape and ateline TS morphology appears to be functionally correlated to both plantigrade locomotion and vertical climbing. However, lorisines share a similar TS morphology and do not use semiplantigrade quadrupedalism. Several shared characteristics of TS by these three groups may help determine which locomotor mode is best correlated with the convergent morphology. First, the large muscle-tendon ratio shared by great apes and lorisines has not been discussed. Second, the larger medial head of gastrocnemius in apes and lorisines has not been functionally correlated to any locomotor characteristic. Finally, the medial expansion of soleus in apes has, as yet, no functional explanation. The force and pressure data discussed earlier may be correlated to these morphologies. 

#### 4.8.5. Muscle-Tendon Ratio

As discussed earlier, chimpanzees and lorisines may have a large muscle-tendon ratio. Additionally, Rauwerdink [192] found that primates that utilize climbing generally have longer fibers than those that do not. Because the muscle-tendon ratio is proportional to fiber length and because fiber length is proportional to muscle excursion [124], it is suggested that chimpanzees and lorisines require longer muscle fibers and greater excursion of the TS muscles than other species. If this is true, one should expect to find quantitative measures that support this hypothesis. Estimated excursion angles of the ankle in the chimpanzee [124] compared to actual measured excursion angles in the macaque [44, 45, 175, 176] are offered as tentative support for this hypothesis. Additionally, lorisines have long TS muscle fibers compared to galagines [99], suggesting that climbing requires greater excursion than quadrupedal walking and leaping. Indeed, Frey [70] suggests that climbing primates have longer muscles than other species. Finally, the spider monkey may require large excursion during vertical climbing [44, 45, 175, 176] and is suggested to have a muscle-tendon ratio similar to the chimpanzee and lorisines. Thus, the role of excursion (as a measure of contact time) and force production to drive the animal up a vertical support may explain the pattern seen in these primates. 

#### 4.8.6. Medial and Lateral Head Asymmetry in Gastrocnemius

Functionally, a larger medial head of gastrocnemius suggests greater force production is required from this head than from the lateral head. Other morphological and kinetic characters appear to support this suggestion. First, many species with a larger medial head of gastrocnemius also exhibit a larger medial femoral condyles (gorilla [193]; human [82]). This feature is associated with greater loading of the knee medially throughout the range of flexion and extension [194]. Greater loading of the knee medially could potentially cause higher medial loading distal to the knee. Such asymmetry in loading would require greater force exerted by medial gastrocnemius to plantarflex the ankle. Second, foot pressure data, discussed earlier, suggest that during arboreal quadrupedalism and bipedalism, greater pressure is borne medially (relative to laterally) [180]. Higher pressure (and force) medially exerted on the foot should require greater force production from the medial gastrocnemius for plantar flexion. If the correlation between greater medial loading and a larger medial head of gastrocnemius is functional, then muscle mass and kinetic data on the gorilla should support this hypothesis. It is also suggested that *Ateles* will exhibit a similar pattern of asymmetry in gastrocnemius and kinetic data. 

#### 4.8.7. Larger Relative Mass of Soleus

It seems reasonable to suggest that in anthropoids, some of the function of plantaris is assumed by soleus because plantaris generally has a significant portion (>5%) of slow-oxidative fibers. In those species where plantaris is absent (e.g., gibbons, orangutans, gorilla, spider monkey, howler monkey), or significantly diminished (e.g., chimpanzees, humans), it is also reasonable to assume that part of the function of plantaris (the part accomplished by slow-twitch fibers) is assumed by soleus. Additionally, Babcock [99] found that soleus, relative to body mass, in galagos scales with positive allometry. If this positively allometric relationship is a pattern found in all primates, it may be expected that larger species will have a relatively larger soleus. On the other hand, Babcock's data show that the lorisine soleus scales with negative allometry, so the positive allometric scaling of soleus in primates may not be universal. The scaling relationship of soleus should be further investigated for a better understanding of the functional significance of a large soleus. 

#### 4.8.8. Medial Expansion of Soleus

As with the larger medial head of the gastrocnemius, the medial expansion of soleus in apes may be functionally related to an arboreal lifestyle with significant amounts of vertical climbing. EMG on human muscles show that during inverted foot postures, medial fibers of soleus are much more active than lateral fibers of soleus [155]. Because soleus is active in inversion in humans, it is hypothesized that soleus activity is similar in other primates. During climbing, apes exhibit highly inverted foot postures in order to grasp the substrate [186, 189] and may therefore require more fibers medially in soleus to accommodate extended inversion of the foot. It is hypothesized that a medial expansion of soleus was needed to accommodate prolonged inversion during arboreal locomotion, specifically vertical climbing. Such a hypothesis could be supported if EMG and kinetic data were available on apes during vertical climbing. Additionally, if vertical climbing is correlated with a medial expansion of soleus, examining atelines and lorisines may yield convergent morphologies and similar kinematics (to great apes). However, Demes and Guenther [195] found that a single morphotype for a locomotor mode should not be expected. In fact, they found that body size plays an important role in morphotype variation [195]. The medial expansion of soleus in great apes may be a result of increased body size in great apes, such that atelines and lorisines may not require a similar medial expansion of soleus. No data have been reported in atelines or lorisines suggesting a medial expansion of soleus. Finally, the medial expansion of soleus could be related to asymmetry in load bearing mediolaterally, as discussed above for gastrocnemius.

In summary, it is suggested that the morphological features of TS shared by nonhuman great apes, atelines, and lorisines are functionally correlated with the demands of vertical climbing. While semiplantigrade locomotion has similar functional demands in some respects, several morphological traits of TS cannot be correlated with plantigrady. Additionally, lorisines do not use semiplantigrade quadrupedalism, but do share a similar TS morphology with humans, great apes, and atelines. 

## 5. Conclusions

Much of the convergent TS morphology of great apes, atelines, and lorisines appears to be related to a shared locomotor mode, and biomechanical data may support this hypothesis. Clearly, more data on the dynamics of vertical climbing need to be collected before definite conclusions can be drawn about the relationship between TS morphology and vertical climbing. If such a relationship does exist, however, the implications for primate locomotor evolution would be twofold.

First, numerous authors have suggested that there is an evolutionary relationship between vertical climbing and bipedalism (e.g., [45, 46, 65, 66, 196–202]), and at the very least, kinematic data on joint angles and EMG recordings support similarities in movements and muscle activity between these two locomotor modes (e.g., [44, 45, 175, 176, 183]). In fact, humans share several TS morphological characters with great apes, atelines, and lorisines, such as a medially expanded soleus, a relatively large soleus, a relatively small plantaris, and a larger medial head of gastrocnemius compared to the lateral head, suggesting that bipedalism and vertical climbing have similar functional requirements of the TS muscles.

Second, if the functional impetus behind these convergent morphologies of TS is similar among nonhuman great apes, atelines, lorisines, *and* humans, then vertical climbing could have been an important locomotor mode that preadapted TS morphology to the functional demands of bipedal locomotion. This suggests a need for further study of the functional morphology of TS during vertical climbing, as the data currently available are sparse and can only hint at answers. Additionally data that would help elucidate the relationship between TS morphology and function should include (1) a broader phylogenetic sample, (2) biomechanical and in vivo functional data, and (3) additional contractile properties of the muscles. Finally, the biomechanical data would be greatly enhanced if they were available during multiple locomotor modes (e.g., plantigrade versus digitigrade versus vertical climbing, etc).

With the addition of more data, hypotheses about the functional morphology of triceps surae could be better addressed. Such data are important for understanding primate locomotion, and for understanding the evolution of the diverse array of locomotor modes seen today. 

## Figures and Tables

**Table 1:** Mean fiber type (±standard deviation, when available) percentages in muscles of selected primates and non-primates.

Species Muscle *(N) *	FG*	FOG*	SO*	Reference
*Felis domesticus*				
LGa (6)	66.65 (4.16)	15.60 (2.46)	17.75 (2.35)	[106]
Mga	42.0	28.0	28.0	[106]
Soleus (6)	0.00	0.78 (0.78)	99.06 (0.94)	[106]
Plantaris				

*Macaca mulatta*				
LGa** (4)		76.00	24.00	[107]
MGa** (4)		78.00	22.00	[107]
Soleus (5)	0.00	39.00 (1.14)	61.00 (0.43)	[108]

*Cavia porcellus*				
LGa (1)	56	32	12	[109]
MGa (1)	54	24	22	[109]
Soleus (1)	0	0	100	[109]
Plantaris (1)	73	23	6	[109]

*Rattus norvegicus*				
LGa (1)	58	37	5	[109]
MGa (1)	58	38	4	[109]
Soleus (1)	0	16	84	[109]
Plantaris (1)	53	41	6	[109]

*Galago senegalensis*				
LGa (1)	56	29	15	[109]
Soleus (1)	0	13	87	[109]
Plantaris	56	30	19	[109]

*Nycticebus coucang*				
LGa (1)	41	14	45	[109]
Soleus (1)	21	7	72	[109]

*Didelphis virginiana*				
LGa (7)	0.00	60.2 (0.4)	38.8 (0.4)	[110]
MGa (7)	0.00	47.4 (0.14)	52.6 (0.14)	[110]

*Macaca fascicularis*				
Deep LGa (3)	49.67	19.67	30.67	[111]
Deep MGa (3)	42.67	28.00	29.33	[111]
Soleus (2)	0.00	6.50	93.50	[111]
Plantaris (3)	48.00	25.33	26.67	[111]

Galago senegalensis***				
LGa (7)	38.00	30.00	25.00	[112]
MGa (7)	40.00	35.00	30.00	[112]
Soleus (7)	0.00	27.00	32.00	[112]
Plantaris (7)	38.00	27.00	23.00	[112]

*Mephitis mephitis*				
LGa (12)	0.00	64.4 (15.1)	35.6 (15.1)	[113]
MGa (12)	0.00	42.7 (4.4)	57.3 (4.4)	[113]
Soleus (12)	0.00	0.00	100.00 (0.0)	[113]
Plantaris (12)		67.2 (6.1)	32.8 (6.1)	[113]

*Mus musculus*				
LGa (12)	69	30	1	[114]
MGa (12)	55	32	8	[114]
Soleus (12)	0	42	58	[114]
Plantaris (12)	41	59	0	[114]

* FG, FOG, and SO fiber types as in Peters et al. [110]. Fiber types correspond to Type IIB, Type IIA, and Type I fibers, respectively. **No distinction was made in the distribution of Type II fibers for this species. Thus, the percentage of Type II fibers was grouped under FOG. ***Subjects had been immobilized for 6 months.

**Table 2:** Relative muscle weight of TS muscles for selected species.

*Species* (N) Muscle	Relative weight as a percentage of total triceps surae mass	Reference
*Homo sapiens* (10)		
LGa	15.63	[67]
MGa	25.77	[67]
Soleus	57.05	[67]
Plantaris	1.55	[67]

*Pan paniscus* (3)		
LGa	19.62	[122, 123]
MGa	28.51	[122, 123]
Soleus	49.84	[122, 123]
Plantaris	2.03	[122, 123]

*Gorilla gorilla* (*3*)		
LGa	20.44	[122]
MGa	32.25	[122]
Soleus	47.30	[122]
Plantaris	NP	[122]

*Pongo pygmaeus* (*2*)		
LGa	19.99	[122]
MGa	34.54	[122]
Soleus	45.47	[122]
Plantaris	NP	[122]

*Pan troglodytes* (3)		
LGa	19.13	[124, 125]
MGa	31.54	[124, 125]
Soleus	48.60	[124, 125]
Plantaris	0.73	[124, 125]

*Hylobates lar* (*3*)		
LGa	26.74	[122, 123]
MGa	36.78	[122, 123]
Soleus	29.46	[123, 126]
Plantaris	7.01	[122, 123]

*Papio spp.* (4)*		
Ga	59.35	[127]
Soleus	29.13	[127]
Plantaris	11.51	[127]

*Macaca fascicularis* (6)		
LGa	35.84	[63, 67, 111]
MGa	30.06	[63, 67, 111]
Soleus	23.83	[63, 67, 111]
Plantaris	10.27	[63, 67, 111]

*Alouatta palliata* (4)		
Ga	54.50	[63]
Soleus	45.50	[63]
Plantaris	NP	[63]

*Alouatta caraya* (2)		
Ga	54.36	[128]
Soleus	45.64	[128]
Plantaris	Variably present	[128]

*Propithecus verreauxi* (1)		
Ga	70.75	[129]
Soleus	10.20	[129]
Plantaris	19.05	[129]

*Avahi laniger* (1)		
Ga	72.31	[129]
Soleus	9.09	[129]
Plantaris	18.60	[129]

*Varecia variegata* (1)		
Ga	65.82	[129]
Soleus	18.99	[129]
Plantaris	15.19	[129]

*Microcebus murinus* (1)		
Ga	70.37	[99]
Soleus	18.52	[99]
Plantaris	11.11	[99]

*Galagoides demidovii* (3)		
Ga	60.0	[99]
Soleus	17.14	[99]
Plantaris	22.86	[99]

*Galago senegalensis* (1)		
Ga	71.11	[129]
Soleus	8.89	[129]
Plantaris	20.00	[129]

*Tarsius syrichta* (1)		
Ga	61.29	[129]
Soleus	19.35	[129]
Plantaris	19.35	[129]

*Galago moholi* (3)		
Ga	67.71	[99]
Soleus	13.54	[99]
Plantaris	18.75	[99]

*Otolemur crassicaudatus* (7)		
Ga	54.43	[99]
Soleus	22.28	[99]
Plantaris	23.28	[99]

*Nycticebus coucang* (9)		
Ga	33.83	[99, 130]
Soleus	59.42	[99, 130]
Plantaris	6.74	[99, 130]

*Nycticebus pygmaeus* (2)		
Ga	38.98	[99]
Soleus	55.93	[99]
Plantaris	5.08	[99]

*Rattus norvegicus* (6)		
Ga	78.42	[131]
Soleus	6.55	[131]
Plantaris	15.02	[131]

*Mus musculus* (19)		
LGa	42.38	[114, 118]
MGa	40.80	[114, 118]
Soleus	7.86	[114, 118]
Plantaris	10.98	[114, 118]

*Oryctolagus cuniculus* (6)		
LGa	41.25	[118]
MGa	25.63	[118]
Soleus	8.12	[118]
Plantaris	25.01	[118]

*Lepus europaeus* (8)		
LGa	28.32	[132]
MGa	31.86	[132]
Soleus	7.52	[132]
Plantaris	32.30	[132]

*Canis familiaris* (6)		
LGa	29.34	[132]
MGa	32.35	[132]
Plantaris	38.31	[132]

*Equus caballus* (7)		
LGa	46.38	[133]
MGa	46.90	[133]
Soleus	0.34	[133]
Plantaris	6.37	[133]

*Loxodonta africana* (4)		
LGa	15.35	[134]
MGa	41.96	[134]
Soleus	22.65	[134]
Plantaris	20.05	[134]

*Ursus maritimus* (1)		
Ga	40	[112]
Soleus	30	[112]
Plantaris	30	[112]

* The baboon sample consists of 2 *Papio anubis* and 2 *Papio hamadryas* [127].

**Table 3:** The three major patterns of mass distribution of triceps surae variation in mammals.

	Pattern 1: gastrocnemius > plantaris > or *≈* soleus	Pattern 2: gastrocnemius > soleus > plantaris	Pattern 3: soleus > or *≈* gastrocnemius > plantaris (if present)
Known genera	*Varecia, Avahi, Propithecus Galagoides, Galago, Otolemur, Tarsius, Rattus, Canis, Mus, Oryctolagus, Lepus, Equus, Loxodonta, Ursus*	Papio, Macaca, Microcebus, Hylobates*	*Homo, Pan, Gorilla, Pongo, Alouatta*

Hypothesized genera	Other non-primate mammals, strepsirrhines other than lorisids	Other cercopithecoids, most platyrrhines (but see pattern three)	Atelines (*Ateles, Lagothrix*), Lorisines

*Hylobates present an interesting case and call into question some of the hypotheses presented in this study.
